# Optimization and prediction of the cotton fabric dyeing process using Taguchi design-integrated machine learning approach

**DOI:** 10.1038/s41598-023-39528-1

**Published:** 2023-07-31

**Authors:** Md. Nahid Pervez, Wan Sieng Yeo, Lina Lin, Xiaorong Xiong, Vincenzo Naddeo, Yingjie Cai

**Affiliations:** 1 Hubei Provincial Engineering Laboratory for Clean Production and High Value Utilization of Bio-Based Textile Materials, Wuhan Textile University, Wuhan, 430200 China; 2 School of Computing, Huanggang Normal University, Huanggang, 438000 China; 3 Sanitary Environmental Engineering Division (SEED), Department of Civil Engineering, University of Salerno, 84084 Fisciano, Italy; 4 Department of Chemical and Energy Engineering, Faculty of Engineering and Science, Curtin University Malaysia, CDT 250, 98009 Miri, Sarawak Malaysia; 5 State Key Laboratory of New Textile Materials and Advanced Processing Technologies, Wuhan Textile University, Wuhan, 430073 China

**Keywords:** Materials science, Structural materials

## Abstract

The typical textile dyeing process calls for a wide range of operational parameters, and it has always been difficult to pinpoint which of these qualities is the most important in dyeing performance. Consequently, this research used a combined design of experiments and machine learning prediction models’ method to offer a sustainable and beneficial reactive cotton fabric dyeing process. To be more precise, we built a least square support vector regression (LSSVR) model based on Taguchi's statistical orthogonal design (L_27_) to predict exhaustion percentage (E%), fixation rate (F%), and total fixation efficiency (T%) and color strength (K/S) in the reactive cotton dyeing process. The model's prediction accuracy was assessed using many measures, including root mean square error (RMSE), mean absolute error (MAE), and the coefficient of determination (R^2^). Principal component regression (PCR), partial least square regression (PLSR), and fuzzy modelling were some of the other types of regression models used to compare results. Our findings reveal that the LSSVR model greatly outperformed competing models in predicting the E%, F%, T%, and K/S. This is shown by the LSSVR model's much smaller RMSE and MAE values. Overall, it provided the highest possible R^2^ values, which reached 0.9819.

## Introduction

Although various synthetic fibers have been developed, cotton remains the world's most significant natural textile fiber because of its exceptional tensile strength, moisture absorption, softness, air permeability, hand feel, and water sorption qualities. The yearly consumption rate of cotton fibers has been breaking records for some time now^1–3^. Cotton also preserves its natural physical qualities despite being subjected to a wide range of dry, wet, and chemical treatments, and it is hydrophilic and alkali resistant. Based on this, cotton fabric has been used widely in producing colored (dyeing) fabrics with respect to its structural characteristics^4, 5^. Reactive dyes have historically been the dyestuff of choice for cotton fiber dyeing due to their many advantages, including excellent colorfastness, repeatability, a broad range of colors, vivid color, convenience, and ease of application. The reactive species in the dye molecules establish covalent connections with the hydroxyl species of the cellulose by nucleophilic substitution or the Michael addition process, resulting in a high color strength property^6–9^.

Conventional reactive dyeing of a cotton fabric involves a series of process parameters salt concentration, dye mass, dye temperature, solution pH, material-to-liquor ratio, and time to achieve a maximum color strength property^10, 11^. Typically, these parameters can substantially impact the amount of chemicals, energy spent, and expenses associated with the dying process throughout the process of dyeing textiles. Most cotton fabric dyeing parameters are based on prior experience rather than a scientific process. Depending on which dyeing parameters are used with a given dyeing formulation, a wide range of performance metrics related to the dyeing process may be attained. The usual approach to process optimization involves modifying one parameter at a time while keeping the others at their current levels. This allows the effect of a single parameter to be studied while keeping the overall optimization intact. On top of that, substantial amounts of time, effort, and experimentation are required. Furthermore, this method may provide inaccurate outcomes, such as the belief that interaction effects cannot be observed, making it difficult to ascertain process parameters' performance^12, 13^. Therefore, process optimization by systematically dyeing cotton fabric in the presence of reactive dyes is a relevant factor in commercial dyeing operations.

With this in mind, a computational tool that can optimize conditions for achieving the desired color strength of the reactive dyed cotton fabric at the lowest production cost may significantly contribute to textile dye assembly, reducing chemical inputs, process time, and economic expenses. In this scenario, the design of experiments, commonly referred to as DOE, is a technique that uses a systematic approach to establish a connection between the factors that affect the outcome of a process and the factors that affect the outcome of the process. The DOE methodology may generally be classified into two distinct categories: the complete factorial design and the Taguchi experimental design. The entire factorial design process involves evaluating and analyzing every potential combination of parameter values. When conducting research using a Taguchi experimental design, one only considers selected levels for assessment. As a result of the use of an orthogonal array (OA) architecture, the Taguchi method is regarded as a reliable approach. The OA can quantitatively determine the appropriate parameters and levels, and it is used to reduce the number of trials, the time of the experiments, the cost, and the quantity of necessary human energy^14–16^. Wahyudin et al.^17^ employed an L9 orthogonal array design using ANOVA analysis as the primary statistical technique to improve the cotton knit fabric dying process, This research confirms that using Taguchi is critical to shortening the re-dyeing stage. According to the research of^2^ a natural dye may be extracted using an L25 Taguchi methodology under ideal circumstances, and this method can then be used to color cotton fabrics. Hossain et al.^18^ adopted a Taguchi design based on an orthogonal array of L9 designs to conduct the deep dyeing of cotton fabric using cacao husk extract to optimize the exhaustion percentage. While this Taguchi model is useful for analyzing data within narrow ranges, it cannot provide reliable predictions for process parameters beyond those limits.

In accelerating the performance of statistical optimization techniques, machine learning (ML) is being used in various fields to speed up these operations and minimize the time and price of simulation. Least square support vector regression models (LSSVRs) are an example of a machine learning model that may get insights directly from data and comprehend how they operate with a wide range of inputs. Most notably, the LSSVR model shows excellent predictive power in determining the expected value of the target output variable in the context of nonlinear data. The LSSVR model is a nonlinear prediction method based on support vector machine theory (SVM). When it comes to decreasing the computational burden associated with reducing the number of viable classes, LSSVR delivers a more efficient response than the SVM by applying a separate set of linear equations in dual space^19, 20^. In recent years, academics from a broad range of disciplines have devoted increasing amounts of attention and interest throughout the course of many years. As such, the revolution of the textile industry 4.0 concept is strongly in line with the adaptation of machine learning so as to maintain sustainability and competence in the market^21^. With the inclusion of ML, textile industries' production, especially dyeing sections, can greatly benefit from human interaction with the interface of intelligent computer-directed tools to enhance production effectiveness. Previous studies have emphasized the importance of ML in the textile industry; for example, Ribeiro et al.^22^ applied automated machine learning in the textile design and finishing features to predict the physical properties of cotton woven fabric. Tsai^23^ underlines sustainable production planning and control strategies using machine learning programming to develop the textile industry 4.0 approach. Similarly, He et al.^24^ used a machine learning-based decision support system for controlling the textile manufacturing process. However, the application of machine learning for the textile dyeing process is rarely reported, which deserves further investigation.

The Taguchi method and machine learning are two different approaches used in various fields. This study uses Taguchi Method to design experiments focusing on parameter optimization and robust design. Even Taguchi based on a table can predict other data, but the Taguchi model is useful for analyzing data within narrow ranges, it cannot provide reliable predictions for process parameters beyond those limits. Hence, this study uses the machine learning model based on Taguchi's statistical orthogonal design to make the prediction. Given it, the present study combined the Taguchi orthogonal model with the LSSVR model for predicting the cotton fabric coloration properties via an industrial dyeing procedure for the first time in the literature. After that, the accuracy of the LSSVR is figured out by calculating the coefficient of determination, also known as R^2^, the root mean square error (RMSE), and the absolute mean error (MAE). In addition, the results are compared to those generated by employing models of the fuzzy model, principal component regression (PCR), and partial least square regression (PLSR) to gain a deeper comprehension of the predictive qualities these models possess.

## Experimental

### Materials and reagents

This study was conducted with 100% pure bleached cotton plain-weave fabric (140 g m^−2^, yarn count 40s Ne) obtained from Jiangnan Group Co., Ltd., China. Commercial dye C. I. Reactive Blue 194 (Table S1) was bought from Shanghai Jiaying Chemical Company, China. The nonionic detergent known as Luton 500 was acquired from the Dalton UK Company. The electrolyte (NaCl, 99.5%) and the alkali (Na_2_CO_3_, 99.8%) arrived from Sinopharm Chemical Reagent Co., Ltd., in Shanghai, China, 200002. Besides these chemicals, all other reagents and chemicals used in the experiment were typically laboratory-quality items.

### Computer-aided dyeing process

#### Taguchi-based optimization approach

**Figure 1:**
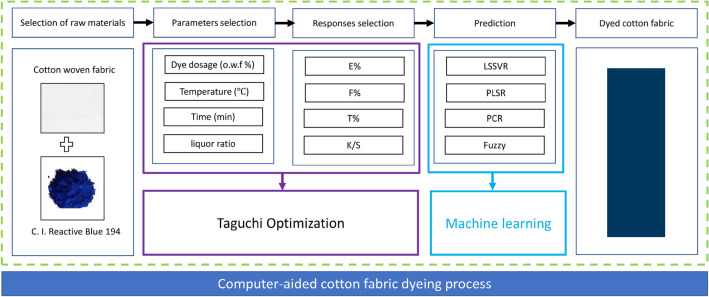
Computer-aided cotton fabric reactive dyeing process.

Before beginning the Taguchi analysis, we established the response functions and the independent, manipulated variables. After that, the number of levels for the independent variables was decided. Minitab®17 statistical software was used to develop Taguchi's orthogonal array-based design of experiments (OA L_27_) (Minitab Inc, Coventry, UK). Tables S2 and S3 provide a detailed overview of the experimental design used to conduct the 27 separate tests across 6 different variables and 3 different factor levels^15^. Dyeing experiments were carried out utilizing a commercial rotary infrared sample dyeing machine (Model HB-HWX24, Ronggui Huibao Dyeing and Finishing Machinery Factory, China) to dye cotton fabric with C. I. Reactive Blue 194 according to the provided design (Fig. 1). The dyebath was prepared by adding sodium chloride at 20 °C, heating, and sodium carbonate. The soaping procedure washed away unfixed dyes. Nonionic detergent (Luton 500, Dalton UK Co.), 2 g L^−1^, was used in the rotary infrared sample dyeing machine's soap washing procedure at a material-to-liquor ratio of 1:10 and a temperature of 95 °C for 15 min (Fig. S1). After being washed with soap, the coloured samples were dried in an oven at 80 °C for 30 min.

#### Machine learning approach

**Figure 2:**
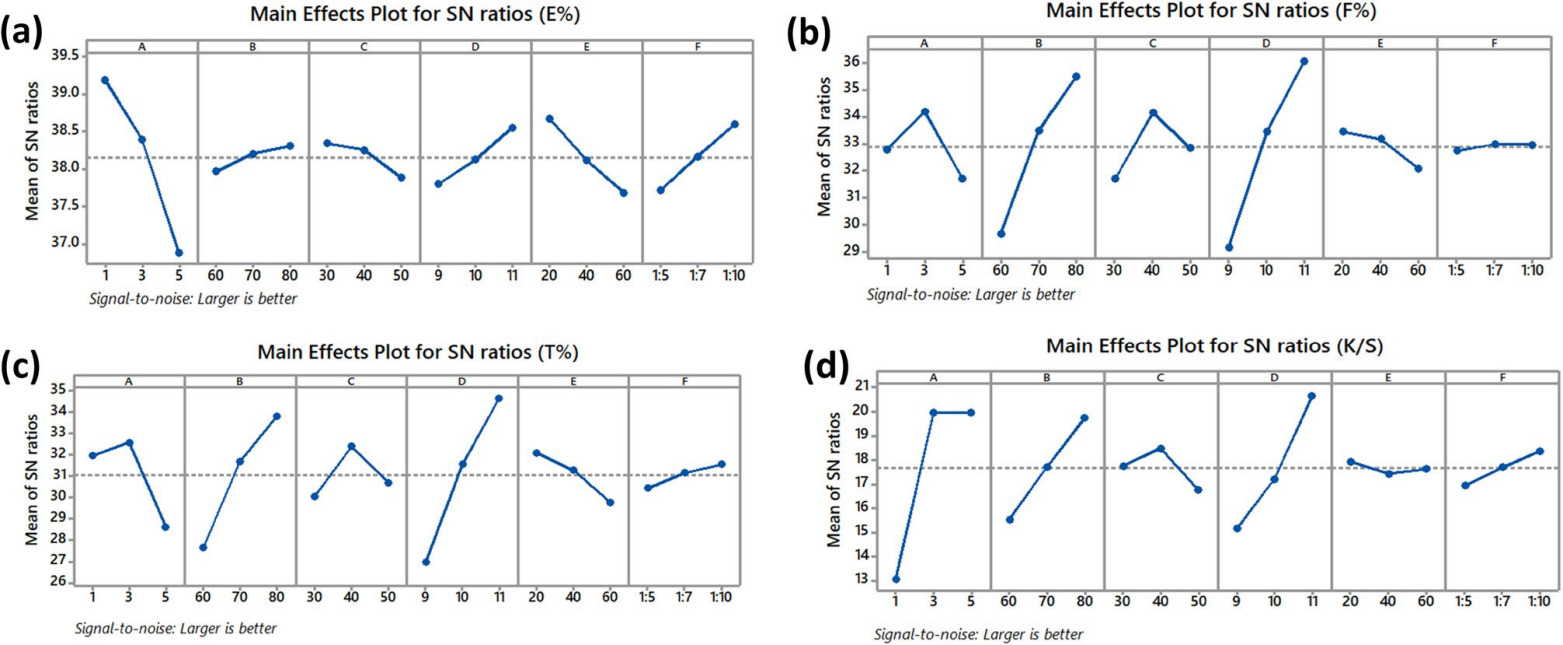
Main effects plot for S/N ratios in (**a**) E%, (**b**) F%, (**c**) T% and (**d**) K/S of B194-dyeings.

A total of 27 datasets were adopted from the dyeing process parameters of cotton fabric. These parameters included the dye dosage, dye-fixing temperature, dye-fixing time, dyebath pH, material-to-liquor ratio, salt concentration, and their responses, which included exhaustion percentage (E%), fixation rate (F%), total fixation efficiency (T%), and color strength (K/S). Once the data was uploaded into MATLAB, it was split 80/20 between the training and testing sets (Table S3). Figure 2 shows the general layout of the four different regression models (PCR, PLSR, Fuzzy approach, and LSSVR) used to predict E%, F%, T%, and K/S values. Models like PCR, PLSR, the fuzzy method, and LSSVR are developed using the training data, as shown in Fig. S2. The models' progress was evaluated using the same models used during training and testing; they included the PCR, PLSR, Fuzzy method, and LSSVR models. The root-mean-square error (RMSE), mean absolute error (MAE), and correlation coefficient (R^2^) values of each available regression model were calculated and compared. A rundown of the many parameter configurations for the PCR, PLSR, Fuzzy approach, and LSSVR models is provided in Table 1. The notations N_T_, N_1_, and N_2_ refer to the total number of datasets used for training, the number of datasets used for testing, and the number of latent variables. Meanwhile, the tuning parameters used in the LSSVR model are represented by the symbols γ, λ, and p. These symbols are referred to as the LSSVR model parameters. The datasets for this cotton fabric dyeing process are available in the supplementary file. Moreover, even though the available models were used, the dataset is still required to train the available models to develop the models, particularly for this cotton fabric dyeing process.

**Table 1:** Values used for PCR, PLSR, Fuzzy method, and LSSVR models.

Parameters	${N}_{T}$	${N}_{1}$	${N}_{2}$	LV	γ	λ	*p*
Values	27	21	6	1	32	0.0625	3.051 × 10^–5^

#### Analysis of prediction behaviour

The root-mean-square error, often known as RMSE, is a scale-dependent defect metric that significantly determines whether a prediction model is effective^25^. Because it allowed comparisons across alternative configurations for a single variable, this metric was used to determine whether or not the data splitting ratio was appropriate. To put it another way, root-mean-square error (RMSE) is a statistic that assesses how far a model strays from the correct answers, with a lower value suggesting that a prediction has a higher degree of accuracy. The RMSE is calculated by taking the root square of the total squared differences between the actual output and the projected output^25^. Alternatively, it might be seen as an indicator of the disparities between the expected values and those observed. When this is considered, a lower RMSE suggests greater accuracy and ability to forecast outcomes. The RMSE formula is shown here as Eq. (1)^26^.

1 $$RMSE = \sqrt {\frac{{\sum\nolimits_{i} {\left( {Y_{i} - \hat{Y}_{i} } \right)}^{2} }}{n}}$$

The $Y_{i}$ shown here signifies the actual output, whereas the $\hat{Y}_{i}$ denotes the predicted output, and n is the total number of samples taken.

The MAE, as shown in Eq. (2), is a statistic that considers neither the directionality nor the severity of errors when evaluating a set of predictions. This value is the weighted mean of the absolute differences between the expected and actual observations for all the test data set observations.

2 $$MAE=\frac{1}{n}{\sum }_{j=1}^{n}|{Y}_{i}-{\widehat{Y}}_{i}|$$

where $\sum {}$ represents the summation.

The coefficient of determination, or R^2^, measures how well a regression model accounts for a certain dataset's variation in the target variable^25^. R^2^ is a statistical measure of the "goodness of fit" between a regression model's observed and expected values. Its worth may range from zero to one^27^. If it's near one, the inputs selected should produce the desired output; if it's farther away, the fit might need some work. The coefficient of determination (R^2^) is found by comparing the sum of squared errors to the sum of squared deviations from the mean of the variable in question. The level of similarity between observed and predicted data is quantified by a statistic called R^2^. Full details of the formula are given in Eq. (3)^28^.

3 $$R^{2} = 1 - \frac{{\sum\nolimits_{i} {\left( {Y_{i} - \hat{Y}_{i} } \right)}^{2} }}{{\sum\nolimits_{i} {\left( {Y_{i} - \overline{Y}} \right)^{2} } }}$$

More specifically, Eq. (4)^29^ provides a mathematical description of the prediction error (PE) that is put to use. We investigate the error of approximation, indicated by Ea, and compute Ea using Eq. (5)^30^ to provide a quantitative demonstration of the predictive abilities of E%, F%, T%, and K/S values.

4 $$PE=\left|\frac{{V}_{1}-{V}_{2}}{{V}_{1}}\right|\times 100\%$$

5 $${E}_{a}=\left(\frac{{N}_{1}}{{N}_{2}}\right){RMSE}_{1}+\left(\frac{{N}_{2}}{N}\right){RMSE}_{2}+\left|{RMSE}_{1}-{RMSE}_{2}\right|$$

where ${V}_{1}$ and ${V}_{2}$ represent the target and actual values, respectively. In training and testing datasets, RMSE_1_ and RMSE_2_ represent the RMSE, while MAE_1_ and MAE_2_ represent the MAE.

### Measurements and characterization

With the use of a UV–visible spectrophotometer (TU-1900 UV–Vis, PERSEE, China), the light absorbance values of the dyebath solution before and after dyeing, as well as the leftover soaping solution, were measured and recorded at the wavelength of maximum absorption, which was 560 nm. Using Eqs. (6)–(8)^18^, we determined the dye exhaustion percentage (E%), fixation rate (F%), and total fixation efficiency (T%).

6 $$E\%=\left(\frac{{A}_{o}-{A}_{t}}{{A}_{o}}\right)\times 100\%$$

7 $$F\%=\left(\frac{{A}_{o}-{A}_{t}-{A}_{s}}{{A}_{o}-{A}_{t}}\right) \times 100\%$$

8 $$T\%=E\%\times F\%\times 100$$

where the light absorbances of the dyebath before dyeing, after dyeing, and the remaining soaping solution is denoted by Ao, At, and As, respectively.

A reflectance spectrophotometer was used to evaluate the color strength, denoted by the K/S ratio, of dyed fabric samples taken randomly from twenty different locations (CHN-Spec CS-650A, Hangzhou Color Spectrum Technology Company, China). The value of the color strength was determined by measuring it at the wavelength at which the dye absorbed the lightest, and the value of the color strength as an average has been presented here.

The surface morphology of the undyed and dyed fabrics was analyzed using Phillips's scanning electron microscope instrument (FE-SEM, Germany). The sample preparation for scanning electron microscopy was carried out as usual. The samples were individually fastened on a standard sample holder after the fabrics were trimmed to sizes no bigger than 1 cm^2^ each. 10 kV was the acceleration voltage, while the working distance was between 15 and 17 mm. A coating of gold was sputtered onto the surfaces of the samples before they were analyzed using SEM. A Nicolet iS5 FT-IR spectroscopy (Thermo Fisher Scientific, USA) was used in order to perform an FTIR study on the undyed and dyed fabrics. In order to investigate the crystallinity behaviour, the X-ray diffraction patterns of the undyed and RB 194-dyed cotton fabric were measured using a Rigaku Ultima III X-ray diffractometer (Tokyo, Japan). The materials were meticulously scissored down to a powdery consistency in preparation for the examination. After that, the powder was placed in a laboratory hydraulic pressing machine and subjected to a static pressure of 127 MPa for sixty seconds to create a circular disc. The sample disc was then put on the holder of the equipment, and the diffraction pattern was recorded with a 2θ angle range from 10 to 80^o^, having a step size of 0.02° under CuKα radiation (λ = 1.54056 Å).

## Results and discussion

### Dyeing process optimization

Process optimization is important in controlling the process to obtain the targeted results. In general, textile dyeing is a complex process equipped with a manufacturing plant function-oriented and requires various steps to maintain the operation performance. According to the Taguchi design (L_27_) (Table S3) in Minitab software, an experiment was conducted to ensure the repetitiveness of the dyeing process. Based on the S/N ratio analysis, optimized parameters for each response, i.e., E% (A_1_B_3_C_1_D_3_E_1_F_3_), F% (A_2_B_3_C_2_D_3_E_1_F_3_), T% (A_2_B_3_C_2_D_3_E_1_F_3_) and K/S (A_3_B_3_C_2_D_3_E_1_F_3_), were found (Fig. 2).

### Characterization

**Figure 3:**
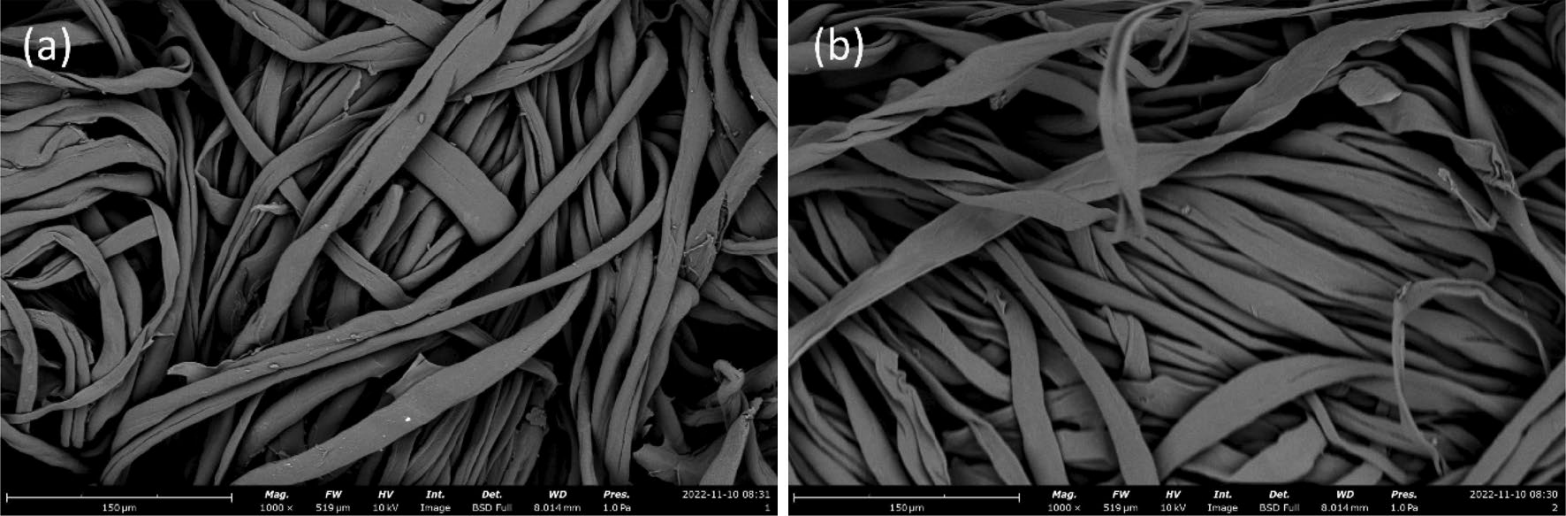
SEM images of undyed fabric (**a**) and (**b**) RB 194 dyed fabric.

The characterization details were measured for the undyed and optimized samples to gain better insights into the dyeing process. The scanning electronic microscope (SEM) was used to investigate the surface morphology of undyed and optimized RB 194-dyed cotton fabrics. The undyed cotton fabric had a seemingly smooth surface patterned with distinctive grooves unique to cotton fibers (Fig. 3a). As a comparison, an SEM image of cotton fabric dyed with RB 194 under optimized conditions showed a similar structure (Fig. 3b), with a smoother surface than the cotton fabric dyed with the undyed sample. This suggests that the dyeing process did not create any discernible alterations in the cotton fabric^31^.

**Figure 4:**
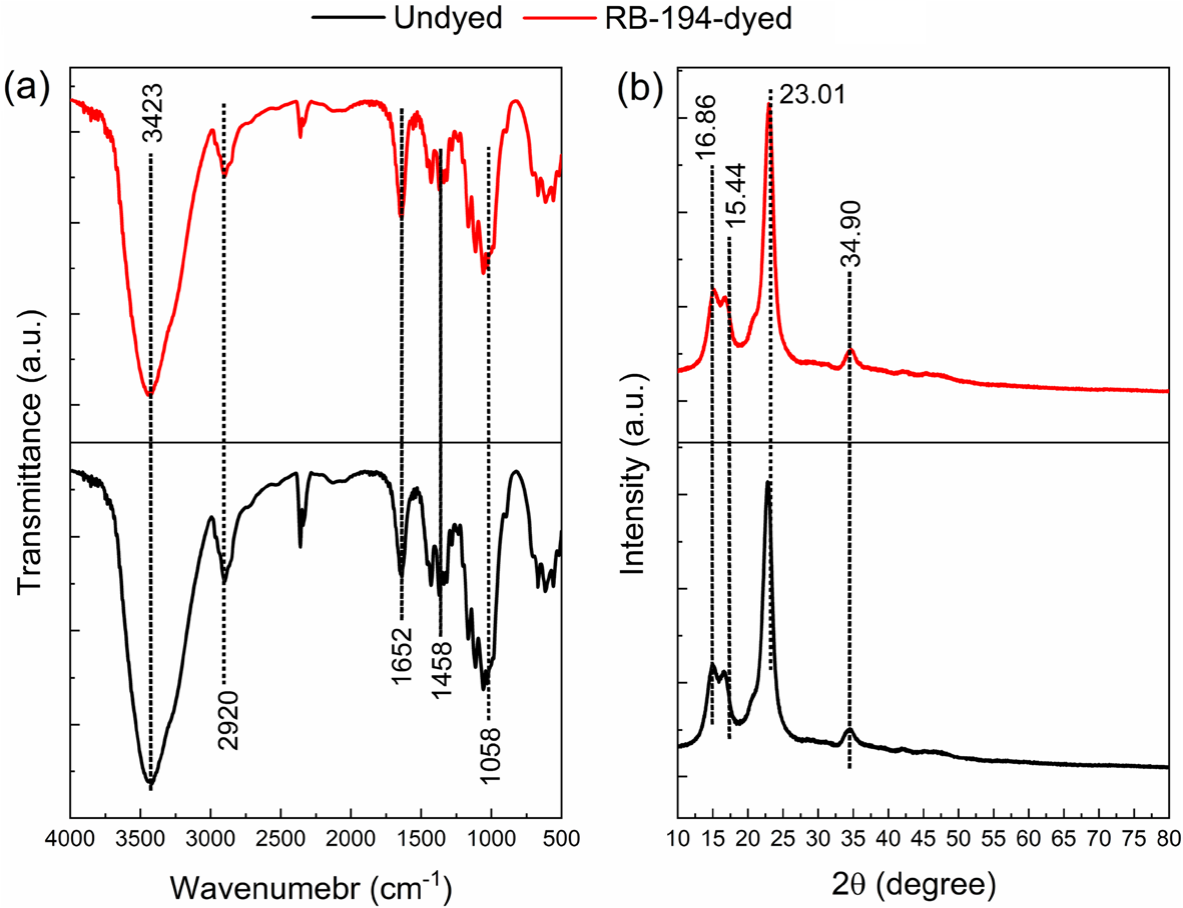
(**a**) FTIR spectra and (**b**) XRD patterns of undyed and RB 194-dyed fabric, respectively.

The variety of functional groups in both the undyed and conditionally optimized RB 194-dyed sample was analyzed using Fourier transform infrared spectroscopy. For the undyed sample (Fig. 4a), a dominating peak at 3423 cm^−1^ may indicate an O–H stretching vibration. Subsequently, the asymmetric stretching vibration of CH_2_ groups may account for a peak of about 2920 cm^−1^. The C=C stretching vibration of aromatic groups corresponds to the absorption peak found at 1652 cm^−1^^32^. The possibility exists that the C–C stretching vibration and the C–H bend stretching vibration are both present in the examined sample since there is a peak at 1458 cm^−1^^33^. The existence of a C–O stretching vibration can be indicated by the peak at 1058 cm^−1^^34^. Considering the optimized conditioned RB 194-dyed sample, the primary characteristic peaks were still intact, and the dyeing procedure did not develop any new peaks, suggesting that the aqueous reactive dyeing medium was appropriate for cotton fabric.

In Fig. 4b, we see XRD patterns for both undyed cotton and RB 194-dyed cotton that has been conditionally optimized. Both samples exhibit the characteristic cellulose I (Iβ) crystalline form, as shown by the 2θ values at 15.44°, 16.86°, 23.01°, and 34.90° in the diffraction pattern^35^. The XRD patterns show that the dyeing procedure did not affect the crystallinity of the cotton. During dyeing, the cellulosic fibers were swollen well, which swelling only occurred in the amorphous zone. Thus, the reactive blue 194 dyes were adsorbed in the amorphous area and the surface of the crystallinity zone. Subsequently, the dyes were fixed with the free hydroxyl groups of cellulosic fiber under the fixation conditions. In order to promote the dyeability of cotton fiber, caustic mercerlization and liquid ammonia treatment^7^ were widely applied because both treatments can damage the crystalline structure of the cotton fiber, i.e., an increase of the amorphous zone, thereby increasing the dye adsorption and fixation rate^36^.

**Figure 5:**
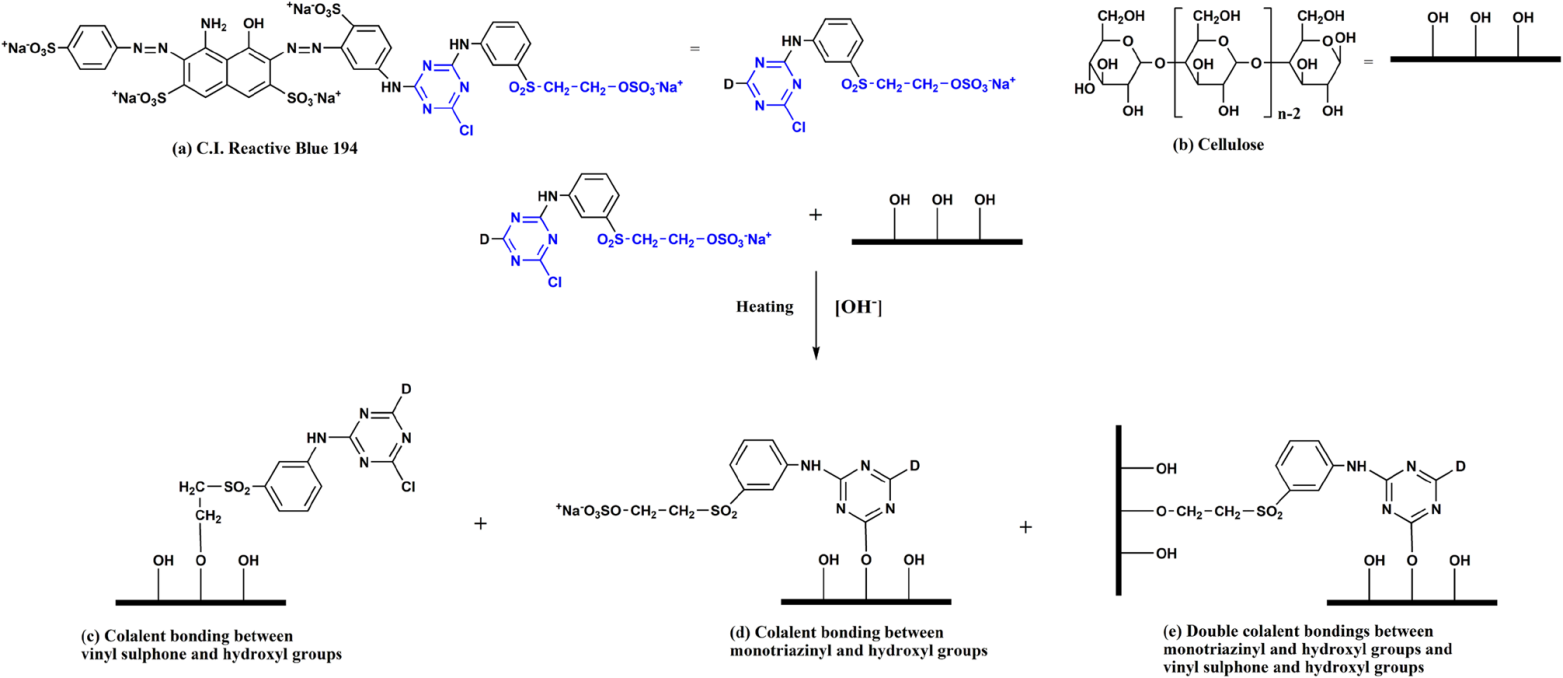
Mechanism of cotton fabric dyeing with RB 194.

The Reactive Blue 194 was adsorbed in cotton fiber and then fixed in cotton fiber via a covalent bond between the reactive group of dye and the hydroxyl group of cellulose after the addition of alkali at the target temperature. The dye fixation mechanism is shown in Fig. 5. Reactive Blue 194 (component a in Fig. 5) is a bifunctional reactive dye with one monochlorotriazinyl group and one vinyl sulphone sulfate; the latter is more reactive than the former^37^. After the addition of alkali, many hydroxyl groups of cellulose (component b in Fig. 5) were changed to cellulosate group (cellulose–O^−^), which is more nucleophilic; meanwhile, the reactive groups became excited. During the dye fixation, the vinyl sulphone sulfate was transferred to the vinyl sulphone group and then reacted with cellulosate to form a covalent bond (component c in Fig. 5). The monochlorotriazinyl group also covalently bonded with cellulosate (component d in Fig. 5). Also, both reactive groups may form covalent bonds with cellulosate (component e in Fig. 5). These covalent bonds contributed to the high performance of the colorfastness to washing dyed cotton fabric^38^.

### Principal component analysis for feature selection

Principal component analysis (PCA) is a dimensional deduction method for unsupervised feature selection based on eigenvectors analysis computing principal components to identify critical original features^39^. In other words, PCA is a method for feature selection that selects variables according to the magnitude (from biggest to smallest in absolute values) of their PCA coefficients or loadings. These coefficients or loadings are the covariances or correlations between the original variables. The larger values denote that a variable has a stronger effect on that principal component and is more important to the corresponding variable^40^. Based on the results from PCA, the variances for the input variables involving the dye dosage, dye-fixing temperature, dye-fixing time, dyebath pH, material-to-liquor ratio, and salt concentration are 276.9231, 69.2308, 69.2308, 2.7692, 0.6923, and 0.0017, respectively. Therefore, the first four input variables, dye dosage, dye-fixing temperature, dye-fixing time, and dyebath pH, are important and capture 99.83% of the variation.

**Figure 6:**
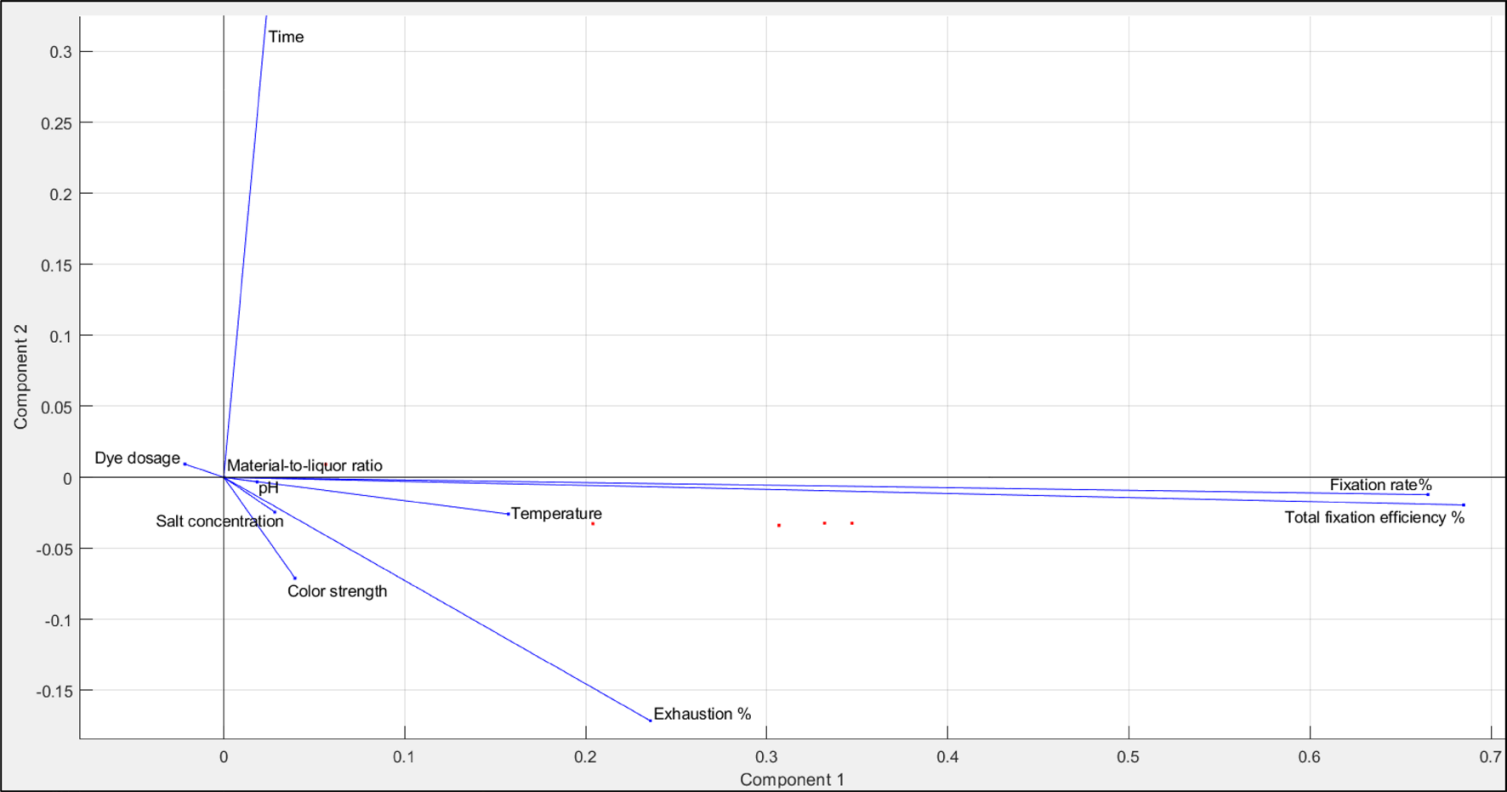
PCA biplot illustrates how strongly each characteristic influences a principal component.

Figure 6 depicts the PCA biplot illustrating how strongly each characteristic influences a principal component. This PCA biplot explores and visualizes the relationship and correlation between the variables^41^. From Fig. 6, it can be seen obviously that the scales of the first four variables, including the dye dosage, dye-fixing temperature, dye-fixing time, dyebath pH, material-to-liquor ratio, and salt concentration, appear relatively higher (have a long distance from the origin). These results are identical to the calculated variances from that PCA in which they indicate that these variables have a significant portion of the variance in the data. Moreover, the principal component scores and loadings for the first two principal components are illustrated in Fig. 6. The axes show the principal component scores, and the vectors are the loading vectors that represent the loading pair per the original variable. It can simply be said that most variables, including the material-to-liquor ratio, pH, temperature, salt concentration, colour strength, and exhaustion percentage, are in the same direction as PC1; hence, they positively correlate with PC1. Meanwhile, the time and dye dosage have the same direction as PC2, indicating they are positively correlated with PC2.

### Modelling assessment

Estimates of the dyed cotton fabric's qualities, such as E%, F%, T%, and K/S values, may be calculated using predictive modelling tools, such as the LSSVR model, combined with the dyeing process parameters. In the past, there have only been a few studies that focused on modelling the dying process within the context of a statistical design^18, 42, 43^. Nevertheless, these predictions can only be made using this method within the bounds of the data that has been supplied. Therefore, in this research, various machine learning models, such as LSVVR, Fuzzy approach, PLSR, and PCR, were developed to overcome the drawbacks of this mathematical modelling methodology by using the characteristics associated with the dyeing process.

The results of the previously described machine-learning models for E%, F%, T%, and K/S, respectively, are summarized in Tables 2, 3, 4, and 5 for convenience. In this work, a model's performance was quantified using three widely used error metrics, including RMSE, MAE, and R2, to exclude any possibility of bias in the evaluation of the model's performance^44^. Tables 2, 3, 4, and 5 show that the training data's error metrics are *RMSE*_*1*_, *MAE*_*1*_, ${{R}_{1}}^{2}$, whereas the testing data's error metrics are *RMSE*_*1*_, *MAE*_*1*_, and ${{R}_{2}}^{2}$.

**Table 2:** Predictive modelling results for E% from LSSVR, Fuzzy method, PLSR, and PCR.

Models	LSSVR	Fuzzy method	PE (%)	PLSR	PE (%)	PCR	PE (%)
Training data							
*RMSE*_*1*_	2.9905	2.2589	24	10.6924	258	10.6125	255
*MAE*_*1*_	2.5067	2.0514	18	9.4379	277	9.3608	273
${{R}_{1}}^{2}$	0.9118	0.9605	5	− 3214.4102	352,620	− 63.7725	7094
Testing data							
*RMSE*_*2*_	5.4307	11.2175	107	9.8863	82	9.6690	78
*MAE*_*2*_	4.9230	9.4050	91	7.0750	44	7.3627	50
${{R}_{2}}^{2}$	0.6606	− 3.6309	650	− 49.3686	7574	− 26.0508	4044
*E*_*a*_	5.9730	13.2083	121	11.3195	90	11.3463	90

**Table 3:** Predictive modelling results for F% from LSSVR, Fuzzy method, PLSR, and PCR.

Models	LSSVR	Fuzzy method	PE (%)	PLSR	PE (%)	PCR	PE (%)
Training data							
*RMSE*_*1*_	2.4614	2.9405	19	16.2657	561	18.3409	645
*MAE*_*1*_	1.8105	2.4981	38	14.9927	728	15.4525	754
${{R}_{1}}^{2}$	0.9813	0.9728	1	− 2.5867	364	− 171.4889	17,575
Testing data							
*RMSE*_*2*_	4.3178	23.3673	441	18.1833	321	23.5394	445
*MAE*_*2*_	3.7186	19.5317	425	17.0150	358	20.6946	457
${{R}_{2}}^{2}$	0.9621	− 7.1373	842	− 0.7999	183	− 10.8179	1224
*E*_*a*_	4.7304	27.9066	490	18.6095	293	24.6947	422

**Table 4:** Predictive modelling results for T% from LSSVR, Fuzzy method, PLSR, and PCR.

Models	LSSVR	Fuzzy method	PE (%)	PLSR	PE (%)	PCR	PE (%)
Training data							
*RMSE*_*1*_	2.5189	2.8530	13	17.2373	584	19.0416	656
*MAE*_*1*_	1.9418	2.5271	30	15.2605	686	16.3155	740
${{R}_{1}}^{2}$	0.9819	0.9773	0	− 3.3742	444	− 145.9199	14,962
Testing data							
*RMSE*_*2*_	5.6166	24.5134	336	18.0975	222	22.9552	309
*MAE*_*2*_	4.6206	20.9400	353	16.2958	253	19.8805	330
${{R}_{2}}^{2}$	0.9359	− 2.9147	411	− 1.0139	208	− 12.0759	1390
*E*_*a*_	6.3049	29.3268	365	18.2886	190	23.8248	278

**Table 5:** Predictive modelling results for K/S from LSSVR, Fuzzy method, PLSR, and PCR.

Models	LSSVR	Fuzzy method	PE (%)	PLSR	PE (%)	PCR	PE (%)
Training data							
*RMSE*_*1*_	0.5174	0.8560	65	3.8945	653	3.6564	607
*MAE*_*1*_	0.3933	0.7457	90	3.4129	768	2.8996	637
${{R}_{1}}^{2}$	0.9815	0.9403	− 4	− 594.6781	− 60,686	− 6.3313	− 745
Testing data							
*RMSE*_*2*_	1.2914	2.8376	120	3.0383	135	3.0535	136
*MAE*_*2*_	1.0123	2.4967	147	2.7166	168	2.0417	102
${{R}_{2}}^{2}$	0.8497	− 5.7888	− 781	− 20.5215	− 2515	− 2.7525	− 424
*E*_*a*_	1.4634	3.2780	124	4.5605	212	4.1252	182

According to Table 2, the LSSVR model performed the best compared to the other models, even though its R^2^ is slightly lower than the fuzzy method for E% in its predictions. The PCR and PLSR models came in second and third, respectively, particularly for those models' training data applications. Compared to the other models, the LSSVR model's RMSE and MAE values are 91% to 107% lower than those of the other models (as shown in Table 2). Consequently, the LSSVR model continues to provide the best overall results. The LSSVR model has R^2^ values that are, except the ${{R}_{1}}^{2}$ value in Table 2, − 650% better than those of the Fuzzy technique, the PCR model, and the PLSR model.

In addition, Table 3 shows that the F% predictions made by the LSSVR model were the most accurate, followed by those made by the Fuzzy technique, the PLSR model, and the PCR model. In Table 3, the LSSVR model has lower RMSE and MAE values (by 19% to 425%) and higher R^2^ (by − 1% to 842%) than the Fuzzy approach, PCR, and PLSR models. Further, Table 4 shows that the LSSVR model, followed by the Fuzzy technique, the PLSR model, and the PCR model, has the best T% prediction accuracy. Table 4 demonstrates that compared to the Fuzzy method, PCR, and PLSR models, the LSSVR model has lower RMSE and MAE values by 13% to 353% and a higher R^2^ by 0% to − 411%. In a similar vein, the LSSVR model fared better than the other models for K/S shown in Table 5 since it had lower RMSE and MAE values (by 65% to 147%, respectively) and a higher R^2^ (by − 4% to − 781%, respectively) than the Fuzzy approach, PCR, and PLSR models.

The LSSVR model outperformed the other models in Tables 2, 3, 4 and 5 because it includes an extra model—the leave-one-out cross-validation model—that finds the optimal tuning parameters for prediction^29^. Furthermore, the nonlinear experimental data is mapped onto a higher dimensional space that may produce a better forecast thanks to the LSSVR model's usage of the renowned kernel function, the radial basis function. Also, besides the LSSVR model, the rest of the models have very low R^2^ values for both training and testing datasets, indicating that these models performed poorly for this cotton fabric dyeing process. The R^2^ values for the LSSVR model are between 0.6606 and 0.9819, which are higher than the benchmarked or acceptable R^2^ value reported by Ozili^45^ and Veerasamy et al.^46^.

Tables 2, 3, 4 and 5 show that the Fuzzy approach implemented in MATLAB's fuzzy logic designer program yields superior results when used on training data. However, the fuzzy technique had the worst results for testing data, with ${{R}_{2}}^{2}$ for E%, F%, T%, and K/S all being negative. This indicates the fuzzy method has a very poor prediction ability. According to Satrio et al.^47^, negative R^2^ values indicate a large gap between the observed and predicted values; in this case, the fuzzy technique in this investigation yielded extremely dissimilar results. This is because the training data creates rules and criteria that form the basis of the fuzzy method's membership function, fuzzy logic operators, and if–then statements^19^. An assortment of fuzzy rules, a database detailing the membership functions used by the fuzzy rules, and a reasoning mechanism outlining the inference path upon the rules to adopt projected data are the three parts of this framework^48^. As a consequence, the fuzzy technique fails to provide satisfactory results if the testing data differs from the training data.

Tables 2, 3, 4 and 5 show that the findings from the PCR and PLSR models are quite comparable, although the PLSR model incorporates both the input and output variables. In contrast, the PCR model only uses the input variables^29, 49^. The similarity in their findings might be attributed to the assumption that the input factors are equally crucial to the prediction accuracy^27^. Both the PCR and the PLSR models were superior to the fuzzy technique when dealing with the testing data, although producing comparable outcomes. This is because PCR and PLSR models are dimension deduction techniques, which means they utilize the training data to fit one or more response variables (predictability variables)^50^. From Tables 2, 3, 4 and 5, notice that there is no condition where the model performed well on the training data but did not perform well on the testing data^51^. Hence, it can be assured that no model is overfitted.

Moreover, from Tables 2, 3, 4 and 5, the Fuzzy method, PLSR, and PCR gave negative R^2^ values, which indicate negative correlations, where the values of one variable tend to increase when the values of the other variable decrease^52^. Also, these negative R^2^ values mean that the Fuzzy method, PLSR, and PCR performed poorly and are unsuitable for the cotton fabric dyeing process.

### Predictivity assessment

**Figure 7:**
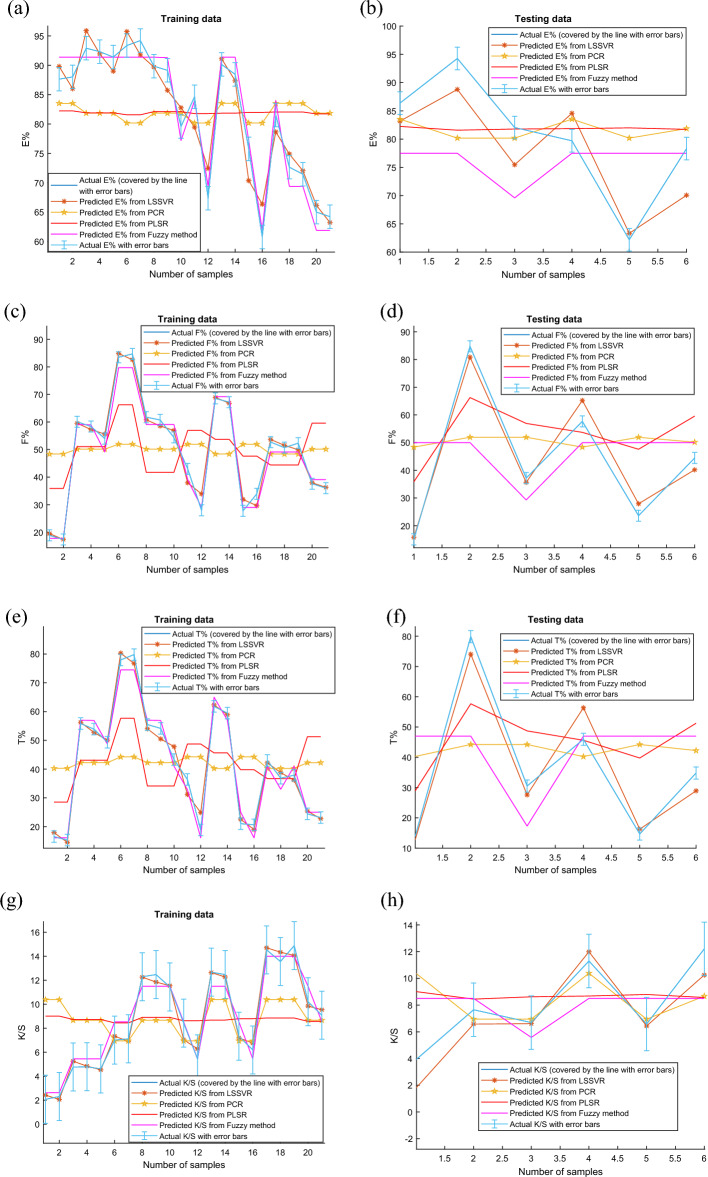
Comparison of predictive results from PCR, PLSR, Fuzzy method, and LSSVR models (**a**) prediction of E% using training data, (**b**) prediction of E% using testing data, (**c**) prediction of F% using training data, (**d**) prediction of F% using testing data, (**e**) prediction of T% using training data, and (**f**) prediction of T% using testing data, (**g**) prediction of K/S using training data, (**d**) prediction of K/S using testing data.

In addition to assessing the model's performance using three error metrics, Fig. 7a–h with error bars for the actual data compare the prediction results for E%, F%, T%, and K/S from the PCR, PLSR, Fuzzy approach, and LSSVR models on the training and testing data. Figure 7a through f show that, in comparison to the fuzzy technique, the PLSR model, and the PCR model, the LSSVR model's projected data is more similar to the actual data. Predictive power increases as the degree of similarity between predicted and observed data decrease^53^. Moreover, from these Fig. 7a–h, it can also be seen that the predicted data from the LSSVR, especially for training data in Fig. 7a,c,e,g are mostly within or close to the error bars. However, the predicted data from the fuzzy technique, the PLSR, and the PCR are outside or far from the error bars. These figures can also conclude that the LSSVR model's superiority in forecasting E%, F%, T%, and K/S is validated beyond a reasonable doubt.

### Accuracy of the models

**Figure 8:**
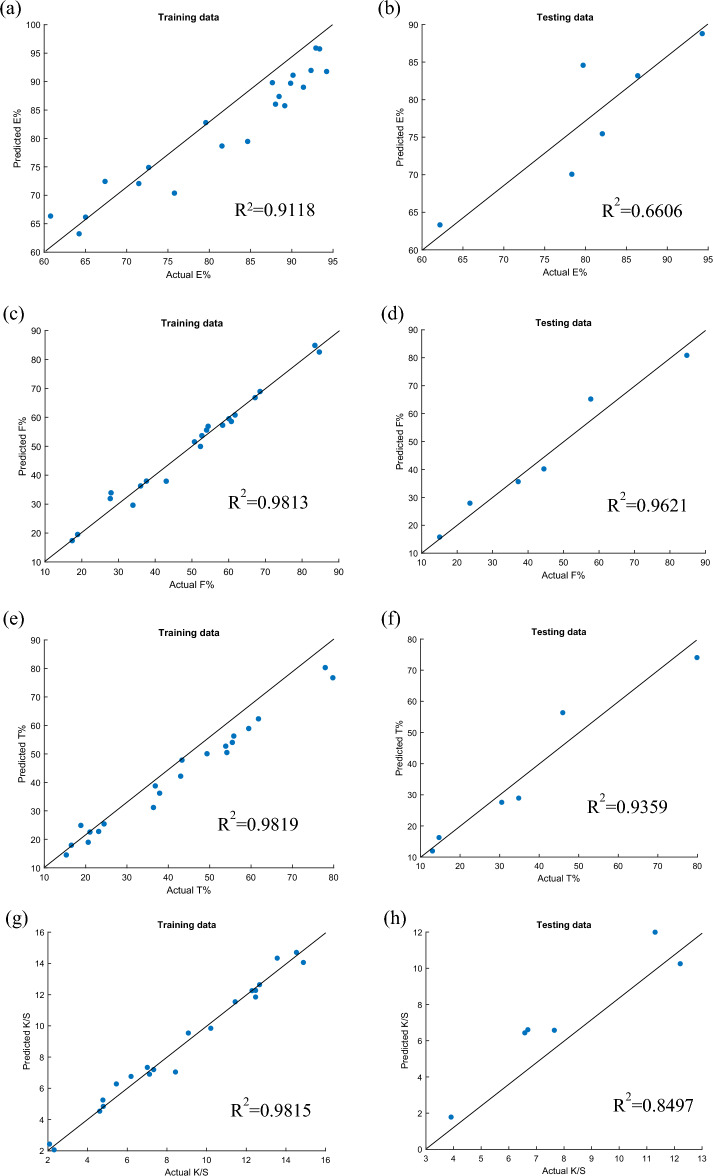
Correlation between the actual and predicted values from the LSSVR model (**a**) E% using training data, (**b**) E% using testing data, (**c**) F% using training data, (**d**) F% using testing data, (**e**) T% using training data, (**f**) T% using testing data. (**g**) K/S using training data, (**h**) K/S using testing data.

The model's correctness in terms of coefficients was also assessed using the same datasets (R^2^). Using both training and test data, the LSSVR model predicts values for E%, F%, T%, and K/S, and these values are compared to the actual values in Fig. 8a–h. The higher the model's predictive ability, the closer the point is to line^54^. Each data point in Fig. 8a through h is rather near the line. This indicates that LSSVR's anticipated outputs are consistent with the tested data for all responses. Good concordance between the model's predicted values and the actual experimental findings is indicative of the model's validity^55^. Important characteristics for optimizing the dyeing performance on cotton fabric, such as E%, F%, T%, and K/S, may be predicted using the LSSVR model.

### Feasibility of sustainable textile industry 4.0

The production of textiles has traditionally required a lot of human work and generated a lot of waste and pollution. The manufacturing process in the textile business used to be quite intricate. A lot of little things needed to be done; therefore, manufacturing tended toward high volume but less variation. According to proponents, "Industry 4.0" ushers in a new era of fully automated, intelligent production. It further incorporates industrial operations systems with information, communication, and intelligence technology. Profitable business models, increased productivity, better quality, and safer working conditions are just some of the many advantages that manufacturing companies may reap from adopting Industry 4.0. Due to these promising implications, it has attracted much interest from academics and professionals^56^.

Emerging in the last several years, both Industry 4.0 and sustainability1 are technical and organizational advancements that are affected by or contribute to increased productivity and environmentally responsible manufacturing. Global competitiveness, fluctuating markets, demand for increasing customization through communication, information, and intelligence, and shrinking innovation and product life cycles are some of the modern difficulties that Industry 4.0 technologies aim to address. Significant opportunities and challenges for sustainable organizational and societal growth are associated with using Industry 4.0 technology. In terms of the bottom line, the advantages of flexible manufacturing include quicker set-up and delivery times, lower labor and material costs, more production flexibility, better productivity, and improved customization^57^.

From a green perspective, energy and resource consumption may be lowered using Industry 4.0 technology for detection and data analysis throughout manufacturing and supply chain operations. Carbon footprint studies that are data-driven and auditable may help reduce waste and greenhouse gas emissions. Disassembling the individual parts of a product is possible by reusing, recycling, or remanufacturing it^58^.

**Figure 9:**
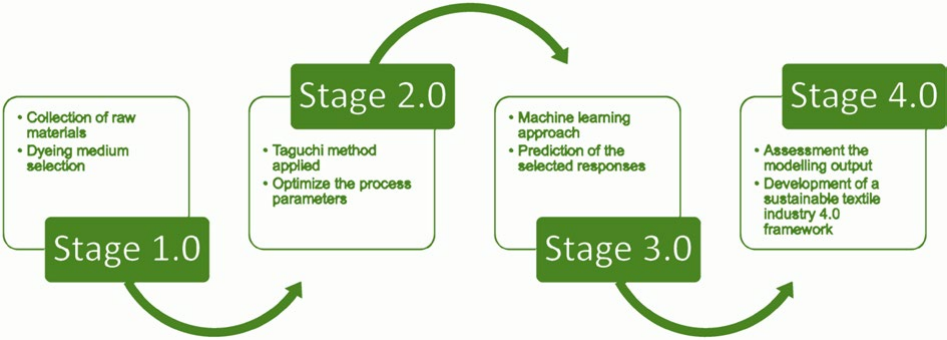
A framework for sustainable textile industry 4.0.

To address the current trend of research, this study employs the Taguchi optimization technique to improve the precision of the dyeing process, in tandem with machine learning to consider the potential constraints of production and impact on attaining maximum security toward sustainable textile industry 4.0 development (Fig. 9).

## Conclusions

The current study developed a novel combined approach LSSVR model, entitled the Taguchi-integrated LSSVR model, for predicting the exhaustion percentage (E%), fixation rate (F%), and total fixation efficiency (T%), and color strength (K/S) of reactive dyed cotton fabric. The experimental results from the dyeing process were used to inform the development of this integrated model, which takes the dye dosage, dye-fixing temperature, dye-fixing time, dyebath pH, material-to-liquor ratio, and salt concentration as input. Finding the optimal circumstances for the dyeing method is crucial for optimizing the cotton fabric's E%, F%, T%, and K/S. Predictive modeling techniques, such as LSSVR, play an important role in the procedures required to produce reactive dyed cotton textiles of the appropriate quality. Compared to the Fuzzy method, PCR, and PLSR models, the LSSVR model performed significantly better in predicting the E%, F%, T%, and K/S, as evidenced by its RMSE and MAE values being 91%–107%, 19% to 425%, 13% to 353% and 65% to 147% lower, respectively, while its R^2^ values were − 650%, − 1% to 842%, 0% to − 411%, and − 4% to − 781% higher, respectively. Results suggest that the LSSVR model might be used as a prediction tool in the textile industry during the dyeing process stage. It was found that the LSSVR model has not been utilized for the cotton fabric dyeing process. Hence, the LSSVR model was introduced in this study. The results of this study show that LSSVR is suitable for the cotton fabric dyeing process, and it is recommended for other similar cases. More study is needed to determine whether or not incorporating another algorithm into the LSSVR model would enhance its prediction ability. It is also important to pay more attention to the new features and expenses connected with improving the dyeing process for cotton textiles as a prospect of the sustainable textile industry 4.0 framework.

## Supplementary Information

Supplementary Information.

## Data Availability

The datasets generated during the current study are available from the corresponding author on reasonable request (Prof. Lina Lin, L. Lin), while the data set is uploaded to https://github.com/pzczxs/MLSSVR.

## Code availability

No code was attempted or used during the current manuscript.

## References

[1] Zhang S (2022). Clean dyeing of cotton fabrics by cationic colored nanospheres. J. Clean. Prod..

[2] Shafiq F (2021). Extraction of natural dye from aerial parts of argy wormwood based on optimized Taguchi approach and functional finishing of cotton fabric. Materials.

[3] Mahmud S, Pervez MN, Taher MA, Mohiuddin K, Liu H-H (2020). Multifunctional organic cotton fabric based on silver nanoparticles green synthesized from sodium alginate. Text. Res. J..

[4] Hossain MY (2021). Adsorption, kinetics, and thermodynamic studies of cacao husk extracts in waterless sustainable dyeing of cotton fabric. Cellulose.

[5] Hossain MY (2022). Green and sustainable method to improve fixation of a natural functional dye onto cotton fabric using cationic dye-fixing agent/D5 microemulsion. J. Nat. Fibers..

[6] Tang AYL, Lee CH, Wang Y, Kan C-W (2022). Polyethylene glycol (PEG) non-ionic surfactant-based reverse micellar dyeing of cotton fabric with hot type trichloropyrimidine (TCP)-based reactive dyes. J. Nat. Fibers..

[7] Lin L (2022). Influence of sequential liquid ammonia and caustic mercerization pre-treatment on dyeing performance of knit cotton fabric. Materials.

[8] Lin L (2022). Combination of pre- and post-mercerization processes for cotton fabric. Materials.

[9] Zhang P (2022). Dyeing of raw ramie yarn with Reactive Orange 5 dye. Ind. Crops. Prod..

[10] Lin L (2022). Sustainable and eco-friendly dyeing of traditional grass cloth with a reactive dye in palm oil medium. RSC. Adv..

[11] Lin L (2021). Combination of wet fixation and drying treatments to improve dye fixation onto spray-dyed cotton fabric. Sci. Rep..

[12] Pervez MN (2017). A novel route to process rationalisation on cellulose dyeing. MATEC Web Conf..

[13] Han L (2022). Short clean dyeing of two-component cotton/polyamide fabrics through adaptive adjustment of the dye solution. J. Clean. Prod..

[14] Shafiq F (2018). Structural relationships and optimization of resin-finishing parameters using the Taguchi approach. Cellulose.

[15] Zhang P (2022). Toward improved performance of reactive dyeing on cotton fabric using process sensitivity analysis. Int. J. Cloth. Sci..

[16] Pervez MN, Shafiq F, Sarwar Z, Jilani MM, Cai Y (2018). Multi-response optimization of resin finishing by using a Taguchi-based grey relational analysis. Materials.

[17] Wahyudin, Kharisma A, Murphiyanto RDJ, Perdana MK, Kasih TP (2017). Application of Taguchi method and ANOVA in the optimization of dyeing process on cotton knit fabric to reduce re-dyeing process. IOP Conf. Ser. Earth Environ. Sci..

[18] Hossain MY (2021). Effluent-free deep dyeing of cotton fabric with cacao husk extracts using the Taguchi optimization method. Cellulose.

[19] Yeo WS, Lau WJ (2021). Predicting the whiteness index of cotton fabric with a least squares model. Cellulose.

[20] Guo Z, Bai G (2009). Application of least squares support vector machine for regression to reliability analysis. Chin. J. Aeronaut..

[21] Ahmad S, Miskon S, Alabdan R, Tlili I (2020). Towards sustainable textile and apparel industry: Exploring the role of business intelligence systems in the era of industry 4.0. Sustainability.

[22] Ribeiro, R. *et al.* In *Artificial Intelligence Applications and Innovations.* (eds Maglogiannis, I., Iliadis, L., & Pimenidis, E.) 244–255 (Springer International Publishing).

[23] Tsai W-H (2018). Green production planning and control for the textile industry by using mathematical programming and industry 4.0 techniques. Energies.

[24] He, Z., Tran, K.-P., Thomassey, S., Zeng, X. & Yi, C. *Developments of Artificial Intelligence Technologies in Computation and Robotics* 550–557.

[25] Neill SP, Hashemi MR, Neill SP, Reza Hashemi M (2018). Ocean modelling for resource characterization. Fundamentals of Ocean Renewable Energy.

[26] Yeo WS, Saptoro A, Kumar P (2019). Adaptive soft sensor development for non-Gaussian and nonlinear processes. Ind. Eng. Chem. Res..

[27] Thien TF, Yeo WS (2022). A comparative study between PCR, PLSR, and LW-PLS on the predictive performance at different data splitting ratios. Chem. Eng. Commun..

[28] Yeo WS, Saptoro A, Kumar P (2020). Missing data treatment for locally weighted partial least square-based modelling: A comparative study. Asia-Pac. J. Chem. Eng..

[29] Yeo, W. S. *2021 International Conference on Green Energy, Computing and Sustainable Technology (GECOST).* 1–5 (IEEE).

[30] Ngu JCY, Yeo C (2022). A comparative study of different kernel functions applied to LW-KPLS model for nonlinear processes. Biointerface. Res. Appl. Chem..

[31] Zhao J (2018). A heterogeneous binary solvent system for recyclable reactive dyeing of cotton fabrics. Cellulose.

[32] Sela SK, Nayab-Ul-Hossain AKM, Rakib MSI, Niloy MKH (2020). Improving the functionality of raw cotton: Simultaneous strength increases and additional multi-functional properties. Heliyon.

[33] Abidi N (2008). Evaluating cell wall structure and composition of developing cotton fibers using Fourier transform infrared spectroscopy and thermogravimetric analysis. J. Appl. Polym. Sci..

[34] Abidi N, Cabrales L, Hequet E (2010). Fourier transform infrared spectroscopic approach to the study of the secondary cell wall development in cotton fiber. Cellulose.

[35] French AD (2014). Idealized powder diffraction patterns for cellulose polymorphs. Cellulose.

[36] Naikwade M, Liu F, Wen S, Cai Y, Navik R (2017). Combined use of cationization and mercerization as pretreatment for the deep dyeing of ramie fibre. Fibers Polym..

[37] Yang Y, Hughes JE (1997). Reactive tendering: The electron-withdrawing inductive effect of reactive dyes on acid hydrolysis of β-1, 4-glucosidic bonds. Textile Chem. Color..

[38] Amin MN, Blackburn RS (2015). Sustainable chemistry method to improve the wash-off process of reactive dyes on cotton. ACS. Sustain. Chem. Eng..

[39] Hasan BMS, Abdulazeez AM (2021). A review of principal component analysis algorithm for dimensionality reduction. J. Soft Comput. Data Min..

[40] de Andrade L (2022). Impact of socioeconomic factors and health determinants on preterm birth in Brazil: A register-based study. BMC Pregnancy Childbirth.

[41] Ferreira E, Macedo E, Fernandes P, Bahmankhah B, Coelho MC (2022). Biplots of kinematic variables and pollutant emissions for an intercity corridor. Transp. Res. Procedia.

[42] Lin L (2022). Sustainable traditional grass cloth fiber dyeing using the Taguchi L16 (4^4^) orthogonal design. Sci. Rep..

[43] Cai Y (2020). Improved reactive dye fixation on ramie fiber in liquid ammonia and optimization of fixation parameters using the Taguchi approach. Dyes Pigments.

[44] Singla P, Duhan M, Saroha S (2021). Review of different error metrics: A case of solar forecasting. AIUB. J. Sci. Eng..

[45] Ozili PK (2023). Social Research Methodology and Publishing Results: A Guide to Non-native English Speakers.

[46] Veerasamy R (2011). Validation of QSAR models-strategies and importance. Int. J. Drug Des. Discov.

[47] Aditya Satrio CB, Darmawan W, Nadia BU, Hanafiah N (2021). Time series analysis and forecasting of coronavirus disease in Indonesia using ARIMA model and PROPHET. Procedia. Comput. Sci..

[48] Kovac P, Rodic D, Pucovsky V, Savkovic B, Gostimirovic M (2013). Application of fuzzy logic and regression analysis for modeling surface roughness in face milliing. J. Intell. Manuf..

[49] Yeo, W. S., Chan, M. K. & Bukhari, N. A. In *International Conference on Intelligent Computing & Optimization.* (eds Vasant, P., Zelinka, I., & Weber, G.W.) 927–937 (Springer, Cham).

[50] Du C, Wei J, Wang S, Jia Z (2018). Genomic selection using principal component regression. Heredity.

[51] Dankers, F., Traverso, A., Wee, L. & van Kuijk, S. (Dekker A., Springer International Publishing, Cham).

[52] Schober P, Boer C, Schwarte LA (2018). Correlation coefficients: Appropriate use and interpretation. Anesth. Analg..

[53] Choi, S., Kim, Y. J., Briceno, S. & Mavris, D. *2016 IEEE/AIAA 35th Digital Avionics Systems Conference (DASC)* 1–6 (IEEE).

[54] Yeo, W. S. & Sung, A. N. *2021 International Conference on Green Energy, Computing and Sustainable Technology (GECOST).* 1–4 (IEEE).

[55] Mesbah M, Soroush E, Rezakazemi M (2017). Development of a least squares support vector machine model for prediction of natural gas hydrate formation temperature. Chin. J. Chem. Eng..

[56] Ghoreishi, M. & Happonen, A. *Proceedings of 6th International Congress on Information and Communication Technology.* (eds Yang, X.-S., Sherratt, S., Dey, N., & Joshi, A.) 189–200 (Springer).

[57] Küsters D, Praß N, Gloy Y-S (2017). Textile learning factory 4.0—Preparing Germany's textile industry for the digital future. Procedia. Manuf..

[58] Majumdar A, Garg H, Jain R (2021). Managing the barriers of Industry 4.0 adoption and implementation in textile and clothing industry: Interpretive structural model and triple helix framework. Comput. Ind..

