# Supplementary material for: Optimization and prediction of the cotton fabric dyeing process using Taguchi design-integrated machine learning approach
Source: Sci Rep. 2023 Jul 31;13:12363. doi: 10.1038/s41598-023-39528-1 (PMC10390507; doi:10.1038/s41598-023-39528-1)
Supplement: Supplementary file 1 — Supplementary Information. [file 41598_2023_39528_MOESM1_ESM.docx]

**Supplementary File**

**Optimization and prediction of the cotton fabric dyeing process using Taguchi design-integrated machine learning approach**

Md. Nahid Pervez ^a,b,c^, Wan Sieng Yeo^d^, Lina Lin^a,e*^, Xiaorong Xiong^b*^, Vincenzo Naddeo^c*^, Yingjie Cai^a^

^a^ Hubei Provincial Engineering Laboratory for Clean Production and High Value Utilization of Bio-based Textile Materials, Wuhan Textile University, Wuhan 430200, China

^b^ School of Computing, Huanggang Normal University, Huanggang 438000, China

^c^ Sanitary Environmental Engineering Division (SEED), Department of Civil Engineering, University of Salerno, Fisciano 84084, Italy

^d^ Department of Chemical and Energy Engineering, Faculty of Engineering and Science, Curtin University Malaysia, CDT 250, 98009 Miri, Sarawak, Malaysia

^e^State Key Laboratory of New Textile Materials and Advanced Processing Technologies, Wuhan Textile University, Wuhan, 430073 China

**^*^ Corresponding authors:**

[linalin@wtu.edu.cn](mailto:linalin@wtu.edu.cn) (L. Lin); [xiongxiaorong@hgnu.edu.cn](mailto:xiongxiaorong@hgnu.edu.cn) (X. Xiong);

[vnaddeo@unisa.it](mailto:vnaddeo@unisa.it) (V. Naddeo).

**Lists of the supplementary materials**

**Figure S1.** Dyeing process curve of C. I. Reactive Blue 194 with cotton fabric

**Figure S2.** A framework of PCR, PLSR, Fuzzy method, and LSSVR models.

**Table S1.** Properties of Reactive Blue 194

**Table S2.** Parameters and their levels

**Table S3.** L27 orthogonal array of factors, experimental data, and S/N ratios

**Table S4.** Dataset for model development

*
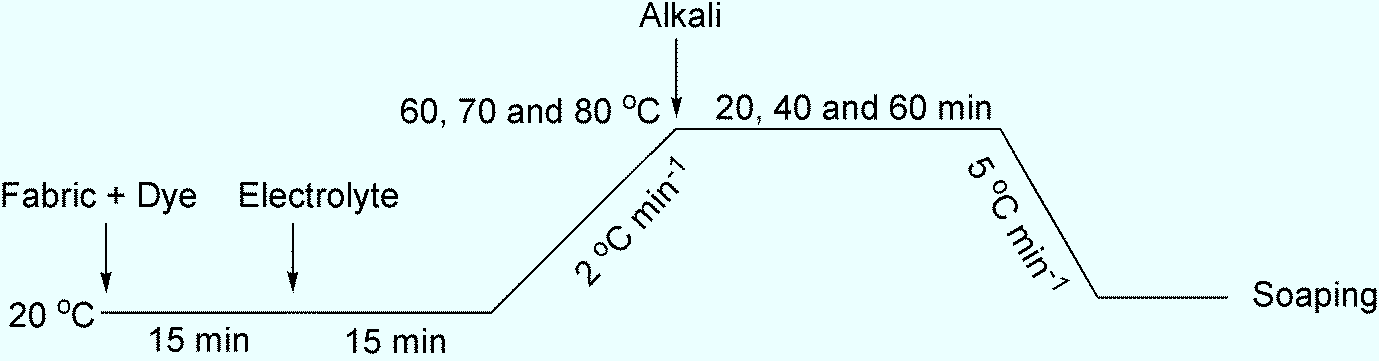
*

**Figure S1.** Dyeing process curve of C. I. Reactive Blue 194 with cotton fabric.


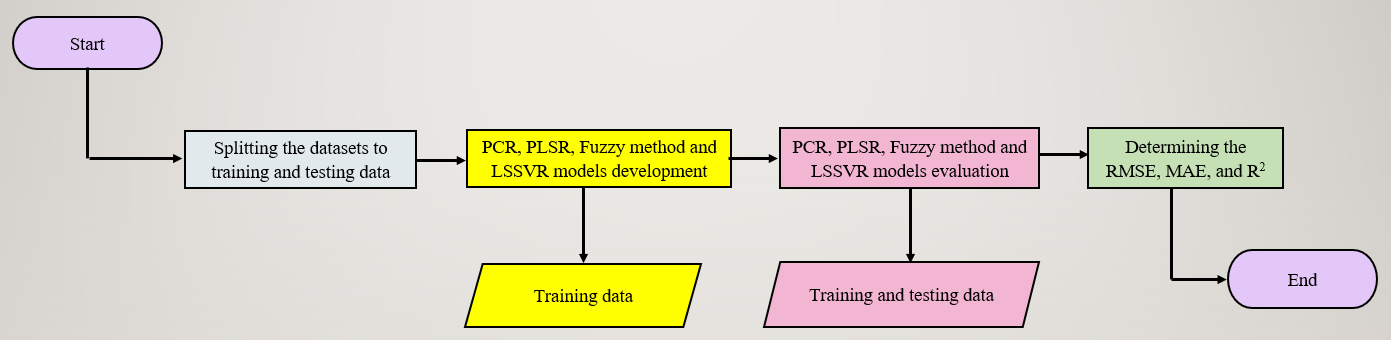


**Figure S2.** A framework of PCR, PLSR, Fuzzy method, and LSSVR models.

**Table S1.** Properties of Reactive Blue 194 (Constructed using JChem and Chemicalize software) ^1^.

| Chemical structure | Molecular Formula | Molecular Weight | Molar refractivity |
| --- | --- | --- | --- |
| 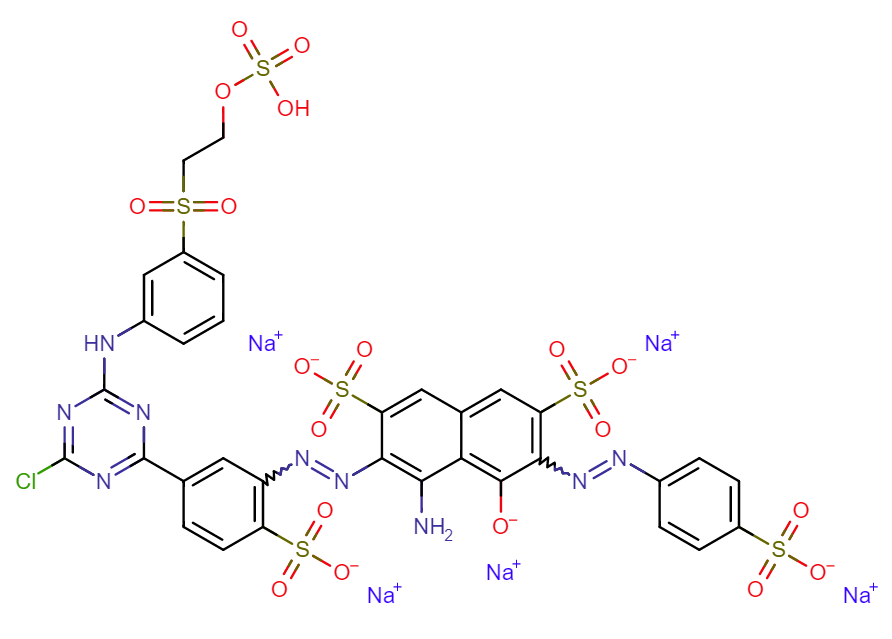 | C_33_H_21_ClN_9_Na_5_O_19_S_6_ | 1190.33 g mol^-1^ | 261.98 cm^3^mol^-1^ |

**Table S2.** Parameters and their levels of the L_27_ orthogonal experimental design

| Factors | |  | Levels | | |
| --- | --- | --- | --- | --- | --- |
| Symbol | **Process parameters** |  | **Level 1** | **Level 2** | **Level 3** |
| A | Dye dosage (o.w.f %) |  | 1 | 3 | 5 |
| B | Dye-fixing temperature (°C) |  | 60 | 70 | 80 |
| C | Salt concentration (g L^-1^) |  | 30 | 40 | 50 |
| D | Dyebath pH |  | 9 | 10 | 11 |
| E | Dye-fixing time (min) |  | 20 | 40 | 60 |
| F | Material-to-liquor ratio |  | 1:5 | 1:7 | 1:10 |

^*^ o. w. f: on the weight of the fabric

**Table S3.** Experimental layout using an (L_27_) orthogonal array, experimental data.

| Exp. No. | Factors | | | | | | Responses - experimental results | | | | | | | |
| --- | --- | --- | --- | --- | --- | --- | --- | --- | --- | --- | --- | --- | --- | --- |
|  | **A** | **B** | **C** | **D** | **E** | **F** | **E%** | **S/N ratio** | **F%** | **S/N ratio** | **T%** | **S/N ratio** | **K/S** | **S/N ratio** |
| 1 | 1 | 60 | 30 | 9 | 20 | 1:5 | 87.65 | 38.85 | 18.87 | 25.51 | 16.54 | 24.37 | 2.08 | 6.36 |
| 2 | 1 | 60 | 30 | 9 | 40 | 1:7 | 88.03 | 38.89 | 17.40 | 24.81 | 15.32 | 23.70 | 2.31 | 7.27 |
| 3 | 1 | 60 | 30 | 9 | 60 | 1:10 | 86.38 | 38.72 | 15.07 | 23.56 | 13.02 | 22.29 | 3.91 | 11.84 |
| 4 | 1 | 70 | 40 | 10 | 20 | 1:5 | 92.92 | 39.36 | 60.08 | 35.57 | 55.83 | 34.93 | 4.77 | 13.57 |
| 5 | 1 | 70 | 40 | 10 | 40 | 1:7 | 92.33 | 39.30 | 58.36 | 35.32 | 53.88 | 34.62 | 4.79 | 13.60 |
| 6 | 1 | 70 | 40 | 10 | 60 | 1:10 | 91.41 | 39.21 | 53.99 | 34.64 | 49.35 | 33.86 | 4.61 | 13.27 |
| 7 | 1 | 80 | 50 | 11 | 20 | 1:5 | 93.37 | 39.40 | 83.48 | 38.43 | 77.95 | 37.83 | 7.01 | 16.91 |
| 8 | 1 | 80 | 50 | 11 | 40 | 1:7 | 94.22 | 39.48 | 84.69 | 38.55 | 79.79 | 38.03 | 7.12 | 17.04 |
| 9 | 1 | 80 | 50 | 11 | 60 | 1:10 | 94.27 | 39.48 | 84.75 | 38.56 | 79.89 | 38.04 | 7.65 | 17.67 |
| 10 | 3 | 60 | 40 | 11 | 20 | 1:7 | 89.86 | 39.07 | 61.74 | 35.81 | 55.48 | 34.88 | 12.29 | 21.79 |
| 11 | 3 | 60 | 40 | 11 | 40 | 1:10 | 89.15 | 39.00 | 60.74 | 35.66 | 54.15 | 34.67 | 12.47 | 21.91 |
| 12 | 3 | 60 | 40 | 11 | 60 | 1:5 | 79.58 | 38.01 | 54.41 | 34.71 | 43.30 | 32.72 | 11.44 | 21.16 |
| 13 | 3 | 70 | 50 | 9 | 20 | 1:7 | 84.65 | 38.55 | 43.00 | 32.66 | 36.40 | 31.20 | 8.43 | 18.51 |
| 14 | 3 | 70 | 50 | 9 | 40 | 1:10 | 82.05 | 38.28 | 37.21 | 31.41 | 30.53 | 29.69 | 6.69 | 16.50 |
| 15 | 3 | 70 | 50 | 9 | 60 | 1:5 | 67.37 | 36.56 | 27.97 | 28.93 | 18.84 | 25.50 | 5.45 | 14.72 |
| 16 | 3 | 80 | 30 | 10 | 20 | 1:7 | 90.15 | 39.09 | 68.55 | 36.72 | 61.80 | 35.81 | 12.67 | 22.05 |
| 17 | 3 | 80 | 30 | 10 | 40 | 1:10 | 88.46 | 38.93 | 67.20 | 36.54 | 59.44 | 35.48 | 12.47 | 21.91 |
| 18 | 3 | 80 | 30 | 10 | 60 | 1:5 | 79.70 | 38.02 | 57.67 | 35.21 | 45.96 | 33.24 | 11.3 | 21.06 |
| 19 | 5 | 60 | 50 | 10 | 20 | 1:10 | 75.78 | 37.59 | 27.76 | 28.86 | 21.04 | 26.46 | 7.33 | 17.30 |
| 20 | 5 | 60 | 50 | 10 | 40 | 1:5 | 60.77 | 35.67 | 33.93 | 30.61 | 20.62 | 26.28 | 6.19 | 15.83 |
| 21 | 5 | 60 | 50 | 10 | 60 | 1:7 | 62.19 | 35.87 | 23.61 | 27.46 | 14.68 | 23.33 | 6.58 | 16.36 |
| 22 | 5 | 70 | 30 | 11 | 20 | 1:10 | 81.54 | 38.22 | 52.71 | 34.43 | 42.98 | 32.66 | 14.53 | 23.24 |
| 23 | 5 | 70 | 30 | 11 | 40 | 1:5 | 72.66 | 37.22 | 50.70 | 34.10 | 36.84 | 31.32 | 13.56 | 22.64 |
| 24 | 5 | 70 | 30 | 11 | 60 | 1:7 | 71.46 | 37.08 | 52.32 | 34.37 | 37.89 | 31.57 | 14.88 | 23.45 |
| 25 | 5 | 80 | 40 | 9 | 20 | 1:10 | 78.32 | 37.87 | 44.46 | 32.95 | 34.82 | 30.83 | 12.21 | 21.73 |
| 26 | 5 | 80 | 40 | 9 | 40 | 1:5 | 65.01 | 36.2596 | 37.61 | 31.50 | 24.45 | 27.76 | 10.21 | 20.18 |
| 27 | 5 | 80 | 40 | 9 | 60 | 1:7 | 64.23 | 36.1548 | 36.03 | 31.13 | 23.14 | 27.28 | 9.08 | 19.16 |

**Table S4.** Dataset for model development

| No. | Input 1 | Input 2 | Input 3 | Input 4 | Input 5 | Input 6 | Output 1 | Output 2 | Output 3 | Output 4 |
| --- | --- | --- | --- | --- | --- | --- | --- | --- | --- | --- |
|  | A | B | C | D | E | F | E% | F% | T% | K/S |
| Training data for model development | | | | | | | | | | |
| 1 | 1 | 60 | 30 | 9 | 20 | 0.2 | 87.65 | 18.87 | 16.54 | 2.08 |
| 2 | 1 | 60 | 40 | 9 | 20 | 0.14 | 88.03 | 17.4 | 15.32 | 2.31 |
| 3 | 1 | 70 | 30 | 10 | 40 | 0.2 | 92.92 | 60.08 | 55.83 | 4.77 |
| 4 | 1 | 70 | 40 | 10 | 40 | 0.14 | 92.33 | 58.36 | 53.88 | 4.79 |
| 5 | 1 | 70 | 50 | 10 | 40 | 0.1 | 91.41 | 53.99 | 49.35 | 4.61 |
| 6 | 1 | 80 | 30 | 11 | 60 | 0.2 | 93.37 | 83.48 | 77.95 | 7.01 |
| 7 | 1 | 80 | 40 | 11 | 60 | 0.14 | 94.22 | 84.69 | 79.79 | 7.12 |
| 8 | 3 | 60 | 40 | 11 | 40 | 0.2 | 89.86 | 61.74 | 55.48 | 12.29 |
| 9 | 3 | 60 | 50 | 11 | 40 | 0.14 | 89.15 | 60.74 | 54.15 | 12.47 |
| 10 | 3 | 60 | 30 | 11 | 40 | 0.1 | 79.58 | 54.41 | 43.3 | 11.44 |
| 11 | 3 | 70 | 40 | 9 | 60 | 0.2 | 84.65 | 43 | 36.4 | 8.43 |
| 12 | 3 | 70 | 30 | 9 | 60 | 0.1 | 67.37 | 27.97 | 18.84 | 5.45 |
| 13 | 3 | 80 | 40 | 10 | 20 | 0.2 | 90.15 | 68.55 | 61.8 | 12.67 |
| 14 | 3 | 80 | 50 | 10 | 20 | 0.14 | 88.46 | 67.2 | 59.44 | 12.47 |
| 15 | 5 | 60 | 50 | 10 | 60 | 0.2 | 75.78 | 27.76 | 21.04 | 7.33 |
| 16 | 5 | 60 | 30 | 10 | 60 | 0.14 | 60.77 | 33.93 | 20.62 | 6.19 |
| 17 | 5 | 70 | 50 | 11 | 20 | 0.2 | 81.54 | 52.71 | 42.98 | 14.53 |
| 18 | 5 | 70 | 30 | 11 | 20 | 0.14 | 72.66 | 50.7 | 36.84 | 13.56 |
| 19 | 5 | 70 | 40 | 11 | 20 | 0.1 | 71.46 | 52.32 | 37.89 | 14.88 |
| 20 | 5 | 80 | 30 | 9 | 40 | 0.14 | 65.01 | 37.61 | 24.45 | 10.21 |
| 21 | 5 | 80 | 40 | 9 | 40 | 0.1 | 64.23 | 36.03 | 23.14 | 9.08 |
| Testing data for model validation | | | | | | | | | | |
| 22 | 1 | 60 | 50 | 9 | 20 | 0.1 | 86.38 | 15.07 | 13.02 | 3.91 |
| 23 | 1 | 80 | 50 | 11 | 60 | 0.1 | 94.27 | 84.75 | 79.89 | 7.65 |
| 24 | 3 | 70 | 50 | 9 | 60 | 0.14 | 82.05 | 37.21 | 30.53 | 6.69 |
| 25 | 3 | 80 | 30 | 10 | 20 | 0.1 | 79.7 | 57.67 | 45.96 | 11.3 |
| 26 | 5 | 60 | 40 | 10 | 60 | 0.1 | 62.19 | 23.61 | 14.68 | 6.58 |
| 27 | 5 | 80 | 50 | 9 | 40 | 0.2 | 78.32 | 44.46 | 34.82 | 12.21 |

| **Notes:** |  |
| --- | --- |
| 1. Liquid ratio was changed to a decimal value (e.g,: a:b is a/b) in the model development and validation because a ratio cannot be used in the simulation. | |
| 2. Noticed that the inclusive liquid ratio has improved the results of the simulation. It should be an important input. | |

**References**

1 ChemAxon. *Free Academic License for JChem*, <Available online: <www.chemaxon.com>>
